# Thyroid surgery in children and young adults: potential overtreatment and complications

**DOI:** 10.1007/s00423-020-01896-x

**Published:** 2020-05-27

**Authors:** Julia I. Staubitz, Julia Bode, Alicia Poplawski, Felix Watzka, Joachim Pohlenz, Hauke Lang, Thomas J. Musholt

**Affiliations:** 1 Section of Endocrine Surgery, Department of General, Visceral and Transplantation Surgery, University Medical Center, Johannes Gutenberg University Mainz, Langenbeckstraße 1, 55131 Mainz, Germany; 2 Department of General, Visceral and Transplantation Surgery, University Medical Center, Johannes Gutenberg University Mainz, Langenbeckstraße 1, 55131 Mainz, Germany; 3 Institute for Medical Biometry, Epidemiology and Informatics, University Medical Center, Johannes Gutenberg-University Mainz, Langenbeckstraße 1, 55131 Mainz, Germany; 4 Section of Pediatric Endocrinology, Center for Pediatrics, University Medical Center, Johannes Gutenberg University Mainz, Langenbeckstraße 1, 55131 Mainz, Germany

**Keywords:** Endocrine, Pediatric thyroid surgery, Papillary thyroid carcinoma follicular thyroid carcinoma

## Abstract

**Purpose:**

Thyroid nodules in the pediatric population are more frequently associated with malignant thyroid disease than in adult cohorts. Yet, there is a potential risk of surgical overtreatment. With this single center study, an analysis of potential overtreatment for suspected malignant thyroid disease in children and young adults was aimed for.

**Methods:**

In a period from 2005 to 2018, 155 thyroid operations in children and young adults performed at the University Medical Center Mainz, Germany, were analyzed (patient age 3–20 years, 117 female). Cases were categorized for preoperative diagnosis: non-malignant (group I, *n* = 45) and malignant thyroid disease (group II, *n* = 110). Postoperative parameters (histology, complication rates) were assessed and compared between groups.

**Results:**

91.1% of group I were histologically benign. 44.5% of group II harbored malignancy. Permanent hypoparathyroidism was documented in group I (2.7%) and in group II (1.4%, *p* = 1.000). Wound infections were absent in group I but observed in group II (0.9%, p = 1.000). Transient vocal cord palsy was recorded only in group I (2.3%, 2/85 vs. 0/177 nerves at risk, *p* = 0.104). Permanent vocal cord palsies were absent.

**Conclusion:**

Preoperative diagnoses were correct in over 90% of group I and in nearly 45% of group II. The high proportion of carcinomas in group II ruled out the issue of potential overtreatment. The risk of severe postoperative complications was equally low in both patient groups.

**Electronic supplementary material:**

The online version of this article (10.1007/s00423-020-01896-x) contains supplementary material, which is available to authorized users.

## Introduction

In children and adolescents, thyroid carcinoma is among the most frequently observed tumor entities of the endocrine system [1]. An increasing incidence was observed over the last decades [2–6]. In 90% of pediatric thyroid carcinoma, papillary thyroid carcinoma (PTC) is the underlying entity, which—in comparison with the adult population—presents more frequently with multifocal disease and metastases to regional neck lymph nodes [7–11]. Follicular thyroid carcinoma (FTC) and poorly differentiated thyroid carcinoma (PDTC) are relatively rare [7]. Medullary thyroid carcinoma (MTC) plays a key role in individuals suffering from multiple endocrine neoplasia (MEN) syndrome, whereas sporadic cases of MTC are infrequent [4, 8, 12]. Even though—compared with the adult population—the literature suggests that thyroid nodules in children and adolescents are more frequently associated with malignant disease [13, 14]; there is yet a risk of surgical overtreatment. Critical evaluation for surgery is required to fulfill the goal phrased by the American Thyroid Association in 2015, that is “to maintain the low disease-specific mortality currently experienced by children with differentiated thyroid carcinoma (DTC)” and “to reduce potential complications resulting from therapy” [4]. Most important complications, which can result from pediatric thyroid surgery, are hypoparathyroidism, vocal cord palsy, or postoperative bleeding. Furthermore, wound infection or lymph fistula may occur.

Apart from careful preoperative selection of patients who should undergo surgery, the choice of the adequate resection strategy is essential. The American Thyroid Association recommends the performance of neck ultrasonography and fine-needle aspiration cytology (FNAC) for the evaluation of pediatric thyroid nodules [15]. However, in adult cohorts, as well as in children and adolescents, FNAC bears limitation for the assessment of follicular lesions and because of the new defined entity “noninvasive follicular thyroid neoplasm with papillary-like nuclear features” (NIFTP) even for papillary thyroid carcinoma [16]. Consequently, the true nature of a “suspicion nodule” remains undetermined in many cases preoperatively [15, 17–23].

This single center study analyzes the spectrum of indications for surgery and performed resection strategies and postoperative outcome in a cohort of children and young adults over a 13-year period. The aim of the study was to answer the questions if there is overtreatment for thyroid nodules (as measured by the number of actual carcinomas in individuals operated for suspected thyroid malignancy), and whether potential overtreatment leads to an increased rate of postoperative complications.

## Material and methods

### Participants

**Fig. 1:**
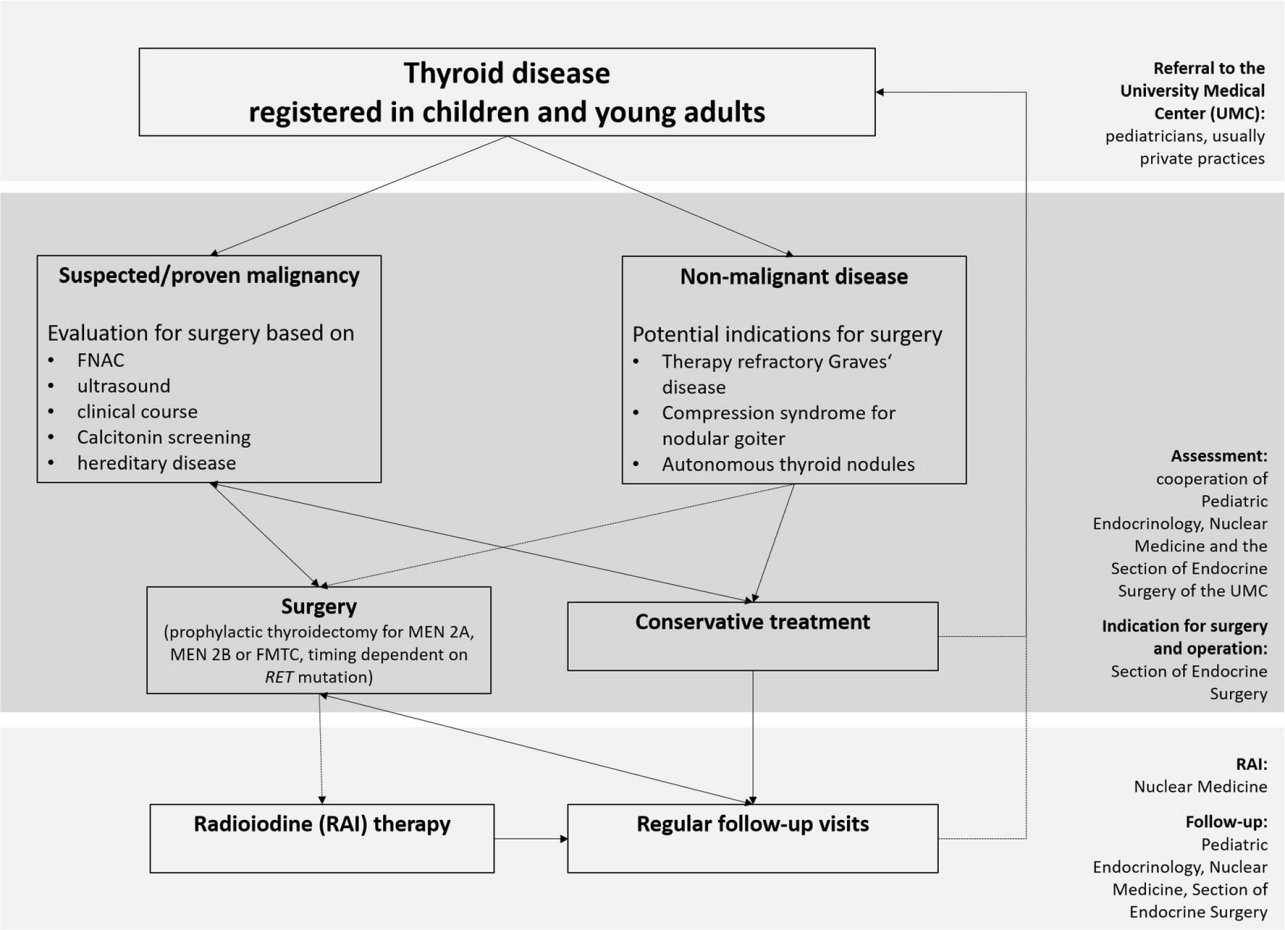
Flow chart: decision-making towards thyroid surgery versus conservative treatment in children and young adults at the University Medical Center Mainz

The study includes all thyroid operations performed in children and young adults at the University Medical Center (UMC) in the period from January 2005 to September 2018. Many children who were examined for thyroid disease and followed in the same time period in our clinic were not included in the study. The evaluation of thyroid nodules included thyroid specific laboratory analysis, repeated neck ultrasound by experienced examiners with assessment of risk factors (microcalcifications, echogenicity, perfusion, margins, diameter, growth, lymph nodes). The TIRADS classification was introduced in 2018 [24, 25]. Suspicious nodules were selected for FNAC, if the result would have impact on the indication of for surgery and/or the operative strategy. FNAC was completed by routine molecular genetic analysis for *BRAF* and *RET/PTC*. In MEN 2 patients, the surgical strategy was based on the preoperative basal calcitonin level. The indication for the surgery was the result of comprehensive diagnostic work-up, as well as the detailed discussion with parents, cooperating pediatricians, endocrinologists, and colleagues from the nuclear medicine department (Fig. 1). All patients underwent routine pre- and postoperative laryngoscopy assessment at the Department of Otolaryngology, UMC Mainz, according to the recommendation of the German Association of Endocrine Surgeons (CAEK) guidelines for thyroid surgery in adults [26–28]. Oncological follow-up was performed as a cooperation between the Department of Pediatrics, Nuclear Medicine, and Surgery Departments.

### Surgery

Although all surgeons of the Section of Endocrine Surgery of the Department of General, Visceral and Transplantation Surgery were involved in the pre- and postoperative patient care, 95.8% of operations were carried by one surgeon (> 19 years expertise in the field of endocrine surgery). Intraoperative frozen section was routinely used in cases with pre- or intraoperative suspected malignancy. Indications for surgery for nonmalignant thyroid disease in children were established according to the CAEK guidelines for the surgical treatment of benign thyroid diseases [28]. Lymph node dissection was performed according to the CAEK practice guidelines for the management of malignant thyroid disease in adults [27] as well as the ATA management guidelines for children with thyroid nodules and differentiated thyroid cancer [7]: lateral lymph node dissection was carried out in case of histologically proven lateral lymph node metastases and central lymph node dissection was performed either in therapeutic or in prophylactic intention, carefully weighing risk of postoperative morbidity with oncological advantages in the individual patient.

### Study design

Cases were grouped according to the preoperative indication for surgery: group I, preoperative non-malignant diagnosis (including Graves’ disease, symptomatic nodular goiter and autonomous thyroid nodules) and group II, preoperative malignant diagnosis (including suspected/preoperatively diagnosed differentiated thyroid carcinoma, suspected/preoperatively diagnosed medullary thyroid carcinoma, completion thyroidectomy for malignancy and prophylactic thyroidectomy in case of hereditary tumor syndromes). Primary endpoints were the postoperative complications transient/permanent vocal cord, transient/permanent hypoparathyroidism, and the necessity of reoperation for bleeding, lymph fistula, or wound infection. Preoperative parameters (FNAC, molecular diagnostics, indication for surgery), intraoperative management, and histological results (primary/secondary diagnosis, TNM classification and oncological outcome) were assessed. Standard postoperative laboratory investigation included parathyroid hormone and serum calcium levels. The postoperative complication “hypoparathyroidism” (parathyroid hormone level below 10 pg/ml) was defined permanent, if persisting for > 6 months postoperatively. Unilateral thyroid operations were excluded from the analysis of postoperative hypoparathyroidism.

### Statistical analysis

Data were documented and described using Microsoft Excel (Microsoft Corporation, Redmond, USA). Median and standard deviation (SD) are presented, when suitable. The description of histological results includes an overview over different age groups (< 6 years, 6–10 years, 10–14 years, 14–16 years, and 16–20 years of age). Further analyses were performed using the IBM Statistical Package for Social Science (SPSS) version 23 (IBM Corporation, Armonk, USA). For postoperative complications, the risk ratio (RR) was calculated, comparing the chance to remain free from complication for the analyzed groups I and II. If procurable, the odds ratio (OR) was additionally calculated. Ninety-five percent confidence intervals (CI) for RR and OR were indicated. Fisher’s exact test was performed to analyze, if preoperative diagnosis and postoperative complications are independent.

## Results

Of a total of 155 operations, 75.5% were performed in female patients (Table 1). Mean patient age was 14 years (range 3–20 years, Table 1). In 11.6%, previous thyroid surgery was registered; 70.6% (12) were carried out in our center. In 14.2%, genetic disorders affecting the thyroid gland were recorded (Table 1). Those were in 40.9% MEN2A, *RET* codon 634 mutation (9), in 18.2% Pendred syndrome (4), in 18.2% *PTEN* hamartoma tumor syndrome, in 9.1% (2) *TPO* gene mutation (hormone synthesis deficiency), in 9.1% MEN2, codon 804 mutation (2) and in 4.5% (1) *DICER1* mutation. Preoperative FNAC was performed in 27.1% of cases (Table 1). There were Bethesda category II (“benign”) in 54.8% (23). Bethesda category V (“suspicious for malignancy”) was diagnosed in 16.7% (7), and Bethesda category IV (“suspicious for follicular neoplasm”) and Bethesda category VI (“malignancy”) were diagnosed in equally 9.5% (4). Independent from the cytological result, a molecular assessment of *BRAFV600E* mutation, as a highly sensitive indicator of the presence of papillary thyroid carcinoma [29], was conducted for 90.5% of FNAC aspirates (38). 10.5% of analyzed cases harbored *BRAFV600E* mutation.

**Table 1:** Descriptive statistics of cohort of children and young adults

	Group 1 (preoperative nonmalignant diagnosis) *n* = 45	Group 2 (preoperative malignant diagnosis) *n* = 110	Total *n* = 155
Age [years] (median ± SD)	15 ± 2.8	14 ± 3.7	15 ± 3.6
Sex ratio female:male (*N*)	40:5	77:33	117:38
Previous thyroid surgery (*N*)	0	18	18
Hereditary thyroid disease (*N*)	2	20	22
Preoperative FNAC (*N*)	1	41	42
Operation time [minutes] (median ± SD)	113 ± 37	107.5 ± 87	109 ± 70
Hospitalization time [days] (median ± SD)	4 ± 3.2	4 ± 1.9	4 ± 2.3
Type of operation			
Total thyroidectomy (*N*)	40	60	100
Subtotal Thyroidectomy (*N*)	0	1	1
Partial Thyroidectomy (*N*)	0	1	1
Lobectomy (*N*)	1	45	46
Lymph node dissection only (*N*)	0	7	7
Lymph node dissection as part of Thyroidectomy(*N*)	1	38	39
Bilateral neck surgery (*N*)	41	72	113
Parathyroid replantation (*N*)	14	47	61
Malignancy in histology (*N*)	4	49	53
Nerves at risk (*N*)	85	177	262
Complications			
Transient hypoparathyroidism (*N*)	14	24	38
Permanent hypoparathyroidism (*N*)	1	1	2
Reoperation for wound infection (*N*)	0	1	1
Reoperation for lymph fistula (*N*)	0	1	1
Transient vocal cord palsy (*N*)	2	0	2

### Surgical management

Mean operation time was 129 (range 28–522 min, Table 1). Mean hospitalization time was 4 days (range 2–13 days). In 64.5%, total thyroidectomy was performed. Lobectomy was carried out in 29.7%. Subtotal and partial thyroidectomies were performed in equally 0.6% (Table 1). Seven patients (4.5%) underwent secondary lymph node dissection following thyroidectomy for persistent lymph node metastases. The overall rate of parathyroid autotransplantation was 39.4% (Table 1). Calculated for bilateral thyroid surgery, this rate was 50.4% (57/113). Autotransplantation of parathyroid glands was necessary, e.g., as a consequence of central lymph node dissection, which, as a part of the operative technique, requires the removal of the lower parathyroid glands. Lymphadenectomy was carried out in 29.0% (46) of cases, in 50.2% (24) as central lymph node dissection (=compartment 1a and 1b [30]). In 17.4% (8), unilateral lateral neck dissection was performed together with central lymph node dissection (=compartment 1a, 1b, and 2 or 3 [30]), and in 2.2% (1) bilateral lateral lymph node resection and central lymph node dissection (=compartment 1a, 1b, 2, and 3 [30]). The remaining cases included selective lymph node dissection (13.0% (6)) and lymph node biopsy for diagnostic reasons (15.2%, 7).

### Histological entities

**Fig. 2:**
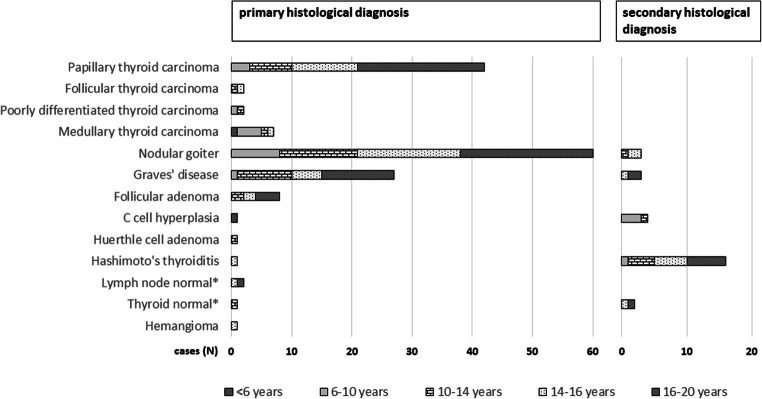
Absolute distribution of final histological diagnoses. Patient age is indicated by bar pattern. The most prevalent primary histological diagnoses were multinodular goiter, papillary thyroid carcinoma and Graves’ disease. Most common associations of primary and secondary histological diagnoses were nodular goiter/Hashimoto’s thyroiditis (12 cases), papillary thyroid carcinoma/Hashimoto’s thyroiditis (4 cases), papillary thyroid carcinoma/Graves’ disease (3 cases) and medullary thyroid carcinoma/C cell hyperplasia (3 cases). * Primary diagnosis: result from completion thyroidectomy

In 34.2% (53/155) of all cases, malignant diagnoses were present in final histology (Fig. 2, Table 2). 62.3% (33) of malignant diagnoses were detected in female patients. 79.2% (42) of malignant diagnoses were histologically PTC (T1a 16.7% (7), T1b 11.9% (5), T2 42.9% (18), T3 23.8% (10), and T4a in 4.8% (2)). Multifocal PTC was diagnosed in 26.2% (11). Lymph node status was distributed as follows: Nx in 11 cases (26.2%), N0 in 13 cases (31.0%), and N1a and N1b in equally 9 cases (21.4%). In 7 of 42 PTC cases, concomitant thyroiditis was diagnosed, 7.1% (3) with Graves’ disease and 9.5% (4) Hashimoto’s thyroiditis. Loco-regional recurrence (lymph node metastases) was registered in one case of PTC.

**Table 2:** Preoperative indication for surgery and postoperative final histology results

	Group 1 (preoperative nonmalignant diagnosis) *n* = 45	Group 2 (preoperative malignant diagnosis) *n* = 110	Total *n* = 155
Indication for surgery			
Graves’ disease	35	0	35
Compression syndrome	7	0	7
Autonomous thyroid nodule	3	0	3
Suspicion for differentiated thyroid carcinoma	0	77	77
Suspicion for medullary thyroid carcinoma	0	2	2
Differentiated thyroid carcinoma (proven)	0	10	10
Prophylactic thyroidectomy	0	10	10
Nodular goiter after chemo-/radiotherapy	0	4	4
Completion thyroidectomy	0	7	7
Postoperative final histology—primary diagnosis			
Benign diagnoses	41	61	102
Nodular goiter	11	49	60
Graves’ disease	27	0	27
Follicular adenoma	3	5	8
Hashimoto’s thyroiditis	0	1	1
Normal lymph node tissue	0	2	2
Normal thyroid tissue	0	1	1
C-cell hyperplasia	0	1	1
Huerthle cell adenoma	0	1	1
Hemangioma thyroid	0	1	1
Malignant diagnoses	4	49	53
Papillary thyroid carcinoma	4	38	42
Follicular thyroid carcinoma	0	2	2
Medullary thyroid carcinoma	0	7	7
Poorly differentiated thyroid carcinoma	0	2	2
Postoperative final histology—secondary diagnosis			
Nodular goiter	2	2	4
Graves’ disease	3	0	3
Hashimoto’s thyroiditis	3	13	16
C-cell hyperplasia	0	4	4
Normal thyroid tissue	0	1	1
No secondary diagnosis	37	90	127

In 13.2% (7), the histological diagnosis was MTC. Six tumors underwent molecular analysis, which in all cases revealed MEN2A Codon 634 mutation. All cases of surgery were the primary operations for these patients. Median of preoperative calcitonin level was 46.8 pg/ml (mean 44.5 ± 21). Postoperatively a stable calcitonin value < 2 pg/ml was achieved in all cases. Among the MTCs, there were 6 pT1a tumors, which were, except for 1 Nx tumor, N0 entities. One tumor was T3 stage, N1b. 50% of hereditary MTC cases had concomitant C-cell hyperplasia (3).

There were 2 cases of FTC (3.8%), one was T2 stage that received completion thyroidectomy after lobectomy. The second case was T1b stage, which was initially treated with thyroidectomy. Both tumors were Nx diagnoses without recurrence. There were two cases of PDTC in the cohort, of which one was treated with lobectomy in the first place, receiving completion thyroidectomy. The other case initially was treated with total thyroidectomy. Recurrence was not registered.

In 65.8%, benign entities were diagnosed (102). 58.8% were nodular goiter (60). In 20.0% (12) of cases with nodular goiter, lymphocytic thyroiditis Hashimoto was recorded as a secondary diagnosis. Graves’ disease was diagnosed in 26.5% (27). C-cell hyperplasia and lymphocytic thyroiditis were diagnosed in equally 1.0% of cases (1). Normal lymph node tissue was registered in two of 102 cases and normal thyroid in one of 102 cases, with the operations being performed as completion resection following previously diagnosed malignant disease.

### Postoperative complications

Median follow-up time was 42 postoperative days (mean 517, range 1–4985 days). The immediate postoperative vocal cord assessment showed a palsy in 0.76% (2/262 nerves at risk (NAR), Table 1). For this analysis, one patient was excluded for preoperative bilateral vocal cord palsy. For two patients, postoperative laryngoscopy was not performed. There were no long-term vocal cord palsies registered. One patient with an early postoperative vocal cord dysfunction did not take part in long-term laryngoscopy control.

Postoperative transient hypoparathyroidism was detected in 33.6% (38/113) of bilateral operations. Permanent hypoparathyroidism was detected in 1.9% (2/106). However, seven patients did not take part in long-term follow-up, of these, six cases with immediate, postoperative hypoparathyroidism.

7.8% (12) of registered cases required a secondary operation. One was for wound infection, one for lymph fistula, and ten for oncologic reasons. Of the latter, eight were completion thyroidectomies, and two were for suspected recurrence of PTC. There were no reoperations for postoperative bleeding.

### Preoperative diagnoses

**Fig. 3:**
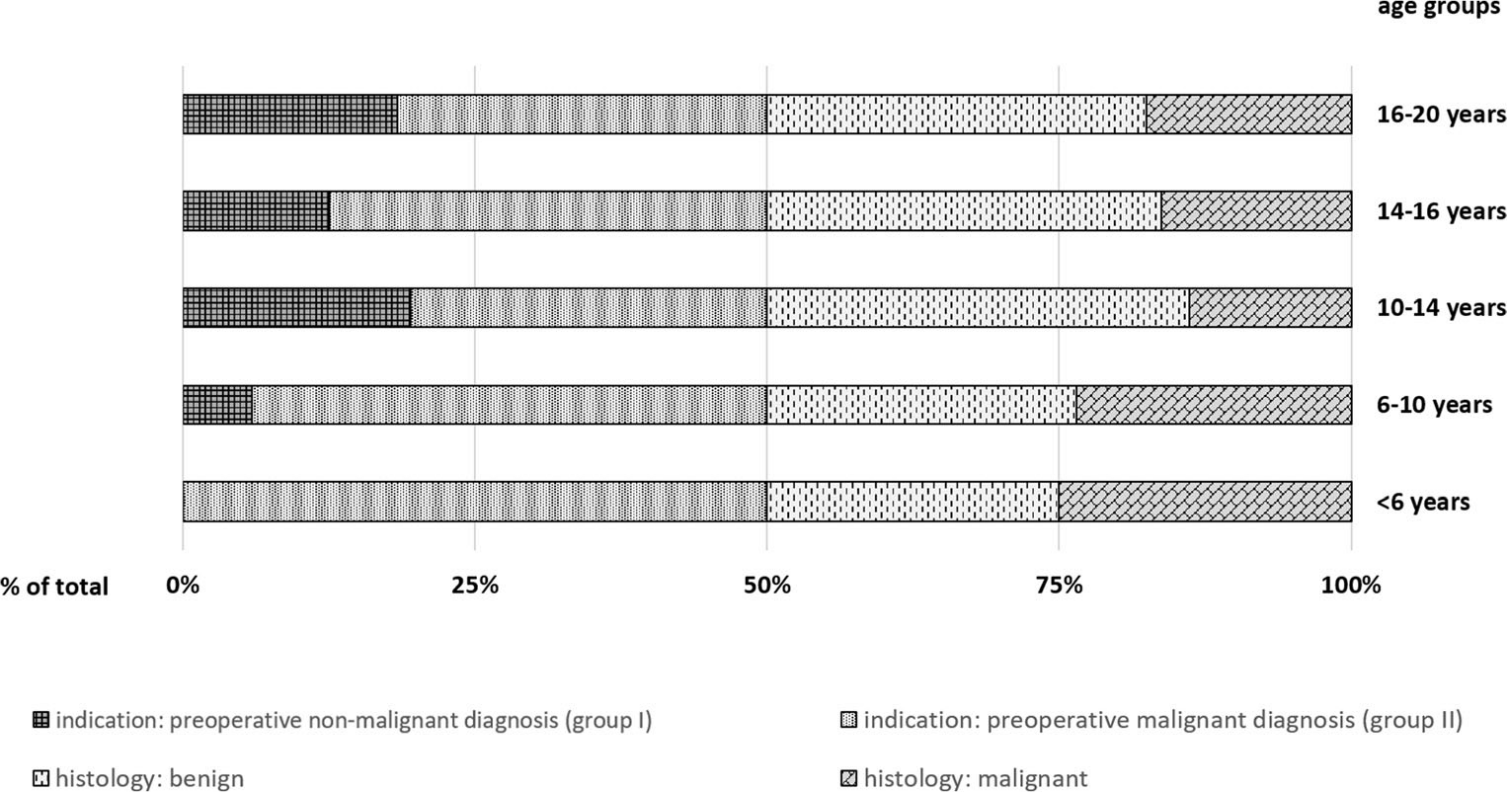
Relative distribution of preoperatively assumed diagnoses and final histological results. In group II, which includes all operations for preoperatively suspected/proven malignancy, final histology revealed the presence of malignant entities in 44.5% of cases (< 6 years 50.0%, 6–10 years 53.3%, 10–14 years 43.5%, 14–16 years 38.7%, 16–20 years 46.2%). In group I, preoperatively established nonmalignant diagnoses were histologically confirmed in 91.1% (6–10 years 100%, 10–14 years 100%, 14–16 years 88.9%, 16–20 years 85.7%)

Nonmalignant diagnoses were established preoperatively in 29.0% (45), referred to as group I (Table 1, Table 2). These were in 77.8% Graves’ disease (35), in 15.6% compression syndrome for nodular goiter (7) and in 6.7% autonomous thyroid nodules (3). Diagnosis of benign thyroid disease was histologically confirmed in 91.1% (41, Fig. 3). Indication for surgery was in 71.0% (110) preoperatively suspected/verified malignant diagnoses (group II). Indications were distributed as follows: 70.0% (77) suspected DTC/intended exclusion of malignancy, 9.1% (10) verified DTC, 9.1% (10) prophylactic thyroidectomy in hereditary disease, 1.8% (2) suspected/verified MTC, 3.6% (4) nodular goiter following radio-chemotherapy, and 6.4% (7) completion surgery. Diagnosis of thyroid carcinoma was confirmed by final histology in 44.5% of cases (49, Fig. 3).

### Postoperative complications in relation to preoperative diagnosis

In group I, transient hypoparathyroidism was present in 34.1% (14/41). In group II, there were 33.3% of transient hypoparathyroidism (24/72, RR 1.012 (0.769–1.332), *p* = 1.000, supplementary Table 1). In group I, there was one case of permanent hypoparathyroidism (1/36, 2.7%). One case was registered with permanent hypoparathyroidism (1/70, 1.4%) in group II. Of group I, five cases of transient hypoparathyroidism were lost to follow-up, and one of group II.

**Fig. 4:**
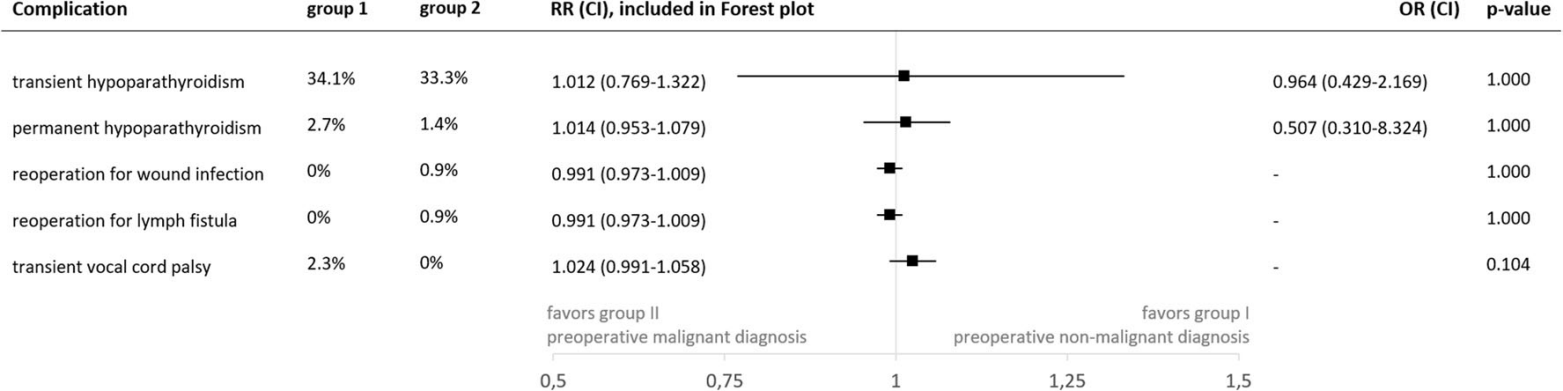
Postoperative complications in relation to preoperative diagnosis. The indication for surgery (group I: preoperative non-malignant diagnosis, group II: preoperative malignant diagnosis) did not significantly influence the occurrence of postoperative complications. Abbreviations: RR R^−^-risk ratio, OR odds ratio, CI 95%-confidence interval

Whereas in group I, reoperation for postoperative complication was not necessary, in group II, reoperation was performed in one case (0.9%) for wound infection (RR 0.991 (0.973–1.009), *p* = 1.000), and also in 0.9% for lymph fistula (RR 0.991 (0.973–1.009), *p* = 1.000, Fig. 4).

Two transient vocal cord palsies were registered in group I (2/85 NAR, 2.3%), whereas in group II, there was none (RR 1.024 (0.991–1.058), *p* = 0.104, Fig. 4). In group I, postoperative laryngoscopy was performed in all cases. One of the two transient vocal cord palsies had recovered in a long-term laryngoscopy control. The second case did not take part in follow-up. Since the intraoperative nerve monitoring verified intact function of the recurrent laryngeal nerve in these cases, a temporary paresis is plausible. For two patients without symptoms of vocal cord palsy, belonging to group II, postoperative laryngoscopy was not performed.

## Discussion

For young children, the literature reported no significant differences in the distribution of thyroid carcinoma between sexes, whereas in young adults, a significant gynaecotropism (5:1) was observed [3–5]. The distribution between the sexes in the present analysis is in line with these results, as the majority of patients diagnosed with thyroid carcinoma was female (62.3%, mean cohort age 14). The overall percentage of malignant entities on final histology was relatively high (34.2%), which can be explained by the preferred referral of patients with strong suspicion for thyroid malignancy and prediagnosed hereditary tumor syndromes to the UMC Mainz. The underlying histological entity was mainly PTC (79.2%, Fig. 2). In group II—comprising operations for suspected/verified thyroid malignancy—final histology revealed the presence of malignant diagnoses in nearly half of all cases (44.5%). This result was consistent in different age subgroups (Fig. 3). The remaining 55.5% of group II harbored benign entities. These include cases of completion thyroidectomy (7 cases) and cases of prophylactic thyroidectomy for hereditary disease (10 cases), preempting the development of malignant disease. From other cohorts reported by specialized institutions operating children for the main indications of suspected malignancy and therapy-refractory Graves’ disease, similar proportions of malignancy were reported as, e.g., in 2011 by Scholz et al. 36% [31] and Wood et al. 37% [32], in 2008 by the Canadian Pediatric Thyroid Nodule (CaPTN) Study Group 43% [33] and in 2019 by Baumgarten et al. 39% [34].

In children and adolescents, complication rates were shown to be higher than in the adult population, with particularly high complication rates at young age < 6 years [35, 36]. An analysis of the Swedish National Registry showed that for total thyroidectomy in children, the rate of permanent hypoparathyroidism reached 7.3% [37]. In the present analysis, median patient age was similar as in the aforementioned study. Yet, permanent hypoparathyroidism was observed only in 1.9%, even though cases of intentional parathyroidectomy for locally advanced thyroid carcinoma were not excluded from analysis. Comparably low rates of permanent hypoparathyroidism were also reported by other specialized centers [31, 32, 38]. Transient hypoparathyroidism was observed in 33.6% of bilateral thyroid operations in the present analysis but recovered in the vast majority of cases (1.9% permanent hypoparathyroidism). In a series of 464 pediatric patients with a similar median patient age of 15 years (36% histologically PTC and 3% MTC), a rate of transient hypoparathyroidism was registered in 37%, and 0.6% with permanent hypoparathyroidism [34]. In the present study, an underlying restoration period following parathyroid autotransplantation appears plausible for this observation. In the literature, children with the diagnosis of Graves’ disease were shown to harbor a higher risk for transient hypoparathyroidism [38]. This study illustrates, that the risk of transient hypoparathyroidism in group I, consisting to 77.8% of cases with therapy refractory Graves’ disease, was similar as in group II, which included operations for preoperatively suspected/verified malignant diagnoses (Fig. 4). These results, however, remain above the 6% of transient hypoparathyroidism documented by Sherman et al. in a cohort of children < 18 years of age suffering from Graves’ disease [39]. Postoperative bleeding requiring secondary surgery did not occur in the present patient cohort, which remains below the incidence of 0.1–2.1%, described in the adult population [40, 41].

11.6% of the present cohort were thyroid reoperations. Thyroid reoperations in the adult population were reported to be associated with transient injury of the recurrent laryngeal nerve in up to 12.5% and permanent lesions in 3.8% [42]. In the present study, only one transient vocal cord palsy (2.3%) was observed during the regularly performed postoperative laryngoscopy. Also, other specialized centers reported similarly favorable results in cohorts of children and adolescents [31, 32].

In this analysis, there were no significant differences in the postoperative complication rates between the patient groups defined by preoperatively established diagnoses. As a consequence, a potential—but yet highly debatable—overtreatment observed in group II did not lead to a significant increase in postoperative complications. Of note is, that in the present study, patients with thyroid malignancy were treated according to the CAEK guidelines [27] and therefore underwent a more radical surgical approach than in other countries: e.g., the ATA management guidelines for children with thyroid nodules and differentiated thyroid cancer allow for near-total thyroidectomy (instead of thyroidectomy) in order to prevent injury of the recurrent laryngeal nerve or parathyroid insufficiency in case of, e.g., papillary thyroid carcinoma [7]. Still, the low complication rate in the present cohort affirms an adequate application of surgical management and was associated with an advantageous oncological outcome in the short-term follow-up (recurrence in only 1 of 53 patients with malignant thyroid disease). Of course, the mean follow-up of 1.4 years does not allow for a meaningful evaluation of disease-free and overall survival since thyroid cancer can recur after long time interval. Both the CAEK and the ATA leave it to the treating surgeons to decide, whether their surgical skills justify the performance of a prophylactic central lymphadenectomy, which potentially contributes to a reduction of redo surgery and an improved disease-free survival [7, 27]. In line with this, for thyroid operations in children, a treatment by specialized high-volume surgeons was recommended in the literature [34–36, 43]. To provide the background for an adequate surgical management, a developed network of pediatric endocrinology, pathology, endocrine surgery, and nuclear medicine holds importance.

## Conclusion

To provide the background for an adequate surgical management of pediatric thyroid disease, the preoperative assessment is of crucial importance, underlining the necessity of a developed network of pediatric endocrinology, pathology, endocrine surgery, and nuclear medicine. Critical indication for surgery allows for a high portion of actual carcinomas in cohorts of children and adolescents undergoing thyroid surgery. Postoperative complications can be kept to a minimum in specialized centers, independent from indication for surgery (malignant vs. nonmalignant thyroid disease).

## Electronic supplementary material

ESM 1 (DOCX 17 kb).
