# Supplementary material for: Thyroid surgery in children and young adults: potential overtreatment and complications
Source: Langenbecks Arch Surg. 2020 May 27;405(4):451–60. doi: 10.1007/s00423-020-01896-x (PMC7359175; doi:10.1007/s00423-020-01896-x)
Supplement: Supplementary file 1 — (DOCX 17 kb). [file 423_2020_1896_MOESM1_ESM.docx]

**Supplementary table 1: Cross tables - postoperative complication for different groups**

| **Complication** | | | | | | |
| --- | --- | --- | --- | --- | --- | --- |
|  | | **Transient hypoparathyroidism** | | | total |  |
|  | | absent | | present |  |  |
| Group | I (not exposed) | 27 | | 14 | 41 |  |
|  | II (exposed) | 48 | | 24 | 72 |  |
|  | | 75 | | 38 | 113 | total |
|  | |  | | | | |
|  | | RR | CI | | p | |
|  | | 1.012 | 0.769-1.332 | | 1.000 | |
|  | | OR |  | |  | |
|  | | 0.964 | 0.429-2.169 | |  | |
| **Complication** | | | | | | |
|  | | **Permanent hypoparathyroidism** | | |  |  |
|  | | absent | | present |  |  |
| Group | I (not exposed) | 35 | | 1 | 36 |  |
|  | II (exposed) | 69 | | 1 | 70 |  |
|  | | 104 | | 2 | 106 | total |
|  | |  | |  |  |  |
|  | | RR | CI | | p | |
|  | | 1.014 | 0.953-1.079 | | 1.000 | |
|  | | OR |  | |  | |
|  | | 0.507 | 0.310-8.354 | |  | |
| **Complication** | | | | | | |
|  | | **Reoperation for wound infection** | | |  |  |
|  | | absent | | present |  |  |
| Group | I (not exposed) | 45 | | 0 | 45 |  |
|  | II (exposed) | 109 | | 1 | 110 |  |
|  | | 154 | | 1 | 155 | total |
|  | |  | | | | |
|  | | RR | CI | | p | |
|  | | 0.991 | 0.973-1.009 | | 1.000 | |
| **Complication** | | | | | | |
|  | | **Reoperation for lymph fistula** | | |  |  |
|  | | absent | | present |  |  |
| Group | I (not exposed) | 45 | | 0 | 45 |  |
|  | II (exposed) | 109 | | 1 | 110 |  |
|  | | 154 | | 1 | 155 | total |
|  | |  | | | | |
|  | | RR | CI | | p | |
|  | | 0.991 | 0.973-1.009 | | 1.000 | |
| **Complication** | | | | | | |
|  | | **Transient vocal cord palsy (NAR)** | | |  |  |
|  | | absent | | present | total |  |
| Group | I (not exposed) | 83 | | 2 | 85 |  |
|  | II (exposed) | 177 | | 0 | 177 |  |
|  | | 260 | | 2 | 262 | total |
|  | |  | | | | |
|  | | RR | CI | | p | |
|  | | 1.024 | 0.991-1.058 | | 0.104 | |
